# Different experimental approaches in modelling cataractogenesis: An overview of selenite-induced nuclear cataract in rats

**DOI:** 10.2478/v10102-010-0005-3

**Published:** 2010-03-29

**Authors:** Zuzana Kyselova

**Affiliations:** Institute of Experimental Pharmacology & Toxicology, Slovak Academy of Sciences, SK-84104 Bratislava, Slovakia

**Keywords:** sodium selenite, nuclear cataract, rats, eye lens, crystallins

## Abstract

Cataract, the opacification of eye lens, is the leading cause of blindness worldwide. At present, the only remedy is surgical removal of the cataractous lens and substitution with a lens made of synthetic polymers. However, besides significant costs of operation and possible complications, an artificial lens just does not have the overall optical qualities of a normal one. Hence it remains a significant public health problem, and biochemical solutions or pharmacological interventions that will maintain the transparency of the lens are highly required. Naturally, there is a persistent demand for suitable biological models. The ocular lens would appear to be an ideal organ for maintaining culture conditions because of lacking blood vessels and nerves. The lens *in vivo* obtains its nutrients and eliminates waste products via diffusion with the surrounding fluids. Lens opacification observed *in vivo* can be mimicked *in vitro* by addition of the cataractogenic agent sodium selenite (Na_2_SeO_3_) to the culture medium. Moreover, since an overdose of sodium selenite induces also cataract in young rats, it became an extremely rapid and convenient model of nuclear cataract *in vivo*. The main focus of this review will be on selenium (Se) and its salt sodium selenite, their toxicological characteristics and safety data in relevance of modelling cataractogenesis, either under *in vivo* or *in vitro* conditions. The studies revealing the mechanisms of lens opacification induced by selenite are highlighted, the representatives from screening for potential anti-cataract agents are listed.

## Introduction

Cataract, the opacification of the lens of the eye, is the leading cause of blindness worldwide – it accounts for approximately 42% of all blindness. Thus more than 17 million people are blind because of cataract and worldwide, 28 000 new cases are reported daily. Approximately 25% of the population over 65 and about 50% over 80 have serious loss of vision because of cataract (Minassian *et al*., 2000). There are an estimated 50 million blind people in the world, and cataracts (opacities of the lens in the eye) are responsible for half of these cases (Johnson and Foster, 2004). In the USA, over 1.2 million cataract operations are performed per year; the costs are over 3.4 billion $ (West, 2000).

For *‘age-related cataracts’*, it is thought (based on twin studies) that the heritability for nuclear and cortical cataracts is around 50% (Hammond *et al*., 2001). *‘Congenital cataracts’* are present at birth indicating pathological changes during embryonic development of the lens. Lens development is the result of a series of inductive processes (Graw, 2003), and one of the most important events is the interaction of the lens placode with the surface ectoderm. ‘*Sugar cataracts*’ were noticed a long time before thorough case observations and medical treatment became available (Robman and Taylor, 2005). These types of cataract are regarded to be associated either with diabetes (diabetic cataract), based on biochemical animal investigations (Fan *et al*., 2009; Kumar *et al*., 2009) as well as clinical and epidemiological studies (Chikamoto *et al*., 2009; Theodoropoulou *et al*., 2010) or galactosemia (Bosh, 2006). Pre-senile development of galactosemic cataract is a consequence of a hereditary disease that results in a defect in, or absence of, galactose-metabolizing enzymes.

At present, the only remedy from cataract is surgical removal of the opaque lens and substitution with a clear one made of synthetic polymers. However, in the UK half of the patients put on waiting lists for operation will die before getting surgery (Minassian *et al*., 2000). In the United States, over 1.3 million cataract operations are performed annually at a cost of 3.5 billion dollars. In developing countries there is simply no sufficient number of surgeons to perform cataract operations. Besides significant costs of operation and possible complications, an artificial lens just does not have the overall optical qualities of a normal lens (Spector, 2000). This is the reason for highly required biochemical solutions or pharmacological intervention that will maintain the transparency of the lens; it is estimated that a delay in cataract formation of about 10 years would reduce the prevalence of visuality disabling cataract by about 45% (Kupfer, 1984). Such a delay would enhance the quality of life for much of the world's older and diabetic population and substantially diminish both the economic burden due to disability and surgery related to cataract.

Hence it remains a significant public health problem, and there is a need for suitable biological models that would test potential anti-cataract agents. The ocular lens would appear to be an ideal organ for maintaining culture conditions because of lacking blood vessels and nerves. The lens *in vivo* obtains its nutrients and eliminates waste products via diffusion with the surrounding fluids. The lens opacification observed *in vivo* can be mimicked *in vitro* by the addition of different cataractogenic agents to the culture medium (Dickerson *et al*., 1995; Saxena et al., 1996; Padival and Nagaraj, 2006; Olofsson *et al*., 2007).

Since an overdose of sodium selenite induces cataract in young rats (Shearer *et al*., 1997), it became an extremely rapid and convenient model of nuclear cataract. Sodium-selenite-induced opacification of lens is widely used for studying the effects of various stresses on the lens, modelling various mechanisms of cataract formation and for screening potential anti-cataract agents (Kinoshita, 1974; Chandra *et al*., 1992; Spector *et al*., 1998; Zigler *et al*., 2003; Gosh and Zigler, 2005; Son *et al*., 2007).

This review will firstly deal with principal anatomical and physiological singularities about eye lens with special emphasis on specific lens proteins – crystallins. Second, the article will mention different experimental approaches applied in cataractogenic research. Third, the main focus of this review will be on selenium (Se) and its salt sodium selenite (Na_2_SeO_3_), their toxicological characteristics and safety data in relevance of modelling cataractogenesis, either under *in vivo* or *in vitro* conditions. The studies revealing the mechanisms of lens opacification induced by selenite will be highlighted, the representatives from screening for potential anti-cataract agents will be listed.

## Lens development, anatomy and physiology

The ocular lens is biconvex, relatively pliable and normally transparent tissue held in suspension by ciliary zonules between the aqueous and the vitreous humors. Its anatomical structure and location coupled with its physical and biochemical characteristics are geared towards maintaining an effective transmission and convergence of the visible frequencies of the electromagnetic spectrum from the environmental objects to the retina, meant for image formation and visual perception. Lens function to converge is also dependent on its pliability and consequent adjustments in its curvature. The lens also acts as an optical filter so that the access of ultraviolet (UV) light to the retina is greatly minimized (Varma, 1991).

**Figure 1:**
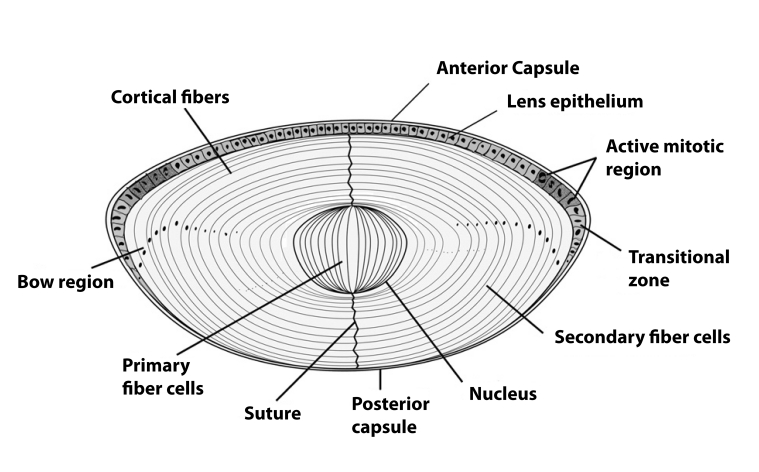
Lens – a single layer of epithelial cells covers the anterior cross section. The elongated fiber cells are in direct contact with the epithelial layer in the anterior region, and they make contact with the capsule in the posterior region. In the lens bow region the cells differentiate, elongate, lose their organelles and begin to form newly differentiated fiber cells (Sharma and Santhoshkumar, 2009).

The eye lens is an avascular tissue encapsulated in a collagenous basement membrane-like material composed of a single layer of epithelial cells on the anterior subcapsular surface (Kuszak, 1990). The lens derives all of its nutrients and oxygen from the aqueous humor and vitreous body. At the equatorial zone the epithelial cells begin to differentiate, elongating to become fiber cells, during which time they lose the organelles and begin synthesizing large quantities of structural proteins called crystallins. This process continues throughout life, though at a slowing down pace, with the younger/newer fiber cells pushing the older fibers to the center (nuclear) region of the lens (Figure 1).

Although the lens has a limited number of cells and cell types, its development is complex (Jaffe and Horwitz, 1990; Lovicu and Robinson, 2004). Lens development begins with the invagination of lens placode toward the optic cup to become the lens pit. The invagination process continues, the pit closes and a lens vesicle is formed in humans by embryonic day 33. Following this, the differentiating epithelial cells start filling the vesicle until the whole cavity is obliterated with fiber cells by the end of the seventh week. The ‘first- formed’ fiber cells that occupy the center of the lens become the embryonic nucleus (Kuszak, 1990; Lovicu and Robinson, 2004) Lens development is not complete without the removal of all potential light-scattering organelles from the fiber cells, which is accomplished in a programmed process that involves proteases (Bassnett, 2002; Bassnett, 2009). The embryonic spherical lens, measuring about 0.35 mm in diameter initially, quickly begins to grow to an elliptical organ of about 35 mg by birth. Studies of human lenses ranging in age from 6 months to 99 years show that the lens actually grows in two phases, an “asymptotic phase” during the prenatal and early childhood periods and a linear phase during the rest of the life span (Jaffe and Horwitz, 1990; Augusteyn, 2007). Although it was once thought that lenses of males are heavier than lenses of females, new data do not support this notion (Augusteyn, 2007). The best estimate of human lens weight can be obtained by the expression:

$$\text{W}=1.38{\text{A}}_{\text{b}}+149\exp\text{ˆ[}\exp\text{ˆ(}1.6-3{\text{A}}_{\text{c}}\text{)],}$$

where W is lens weight in mg, A_b_ is postnatal age in years and A_c_ is the time since conception in years (Augusteyn, 2007). Because the lens is composed of a range of fibers representing different ages, it is an attractive tissue for studying the effects of aging on protein structure and function (Bloemendal, 1981).

There is little protein turnover in the lens, the majority of proteins consisting of long-lived α-, β-, γ-crystallins. These proteins appear to be specific to the lens, although they contain regions of sequence and structural homo-logy comparable to other proteins. Lipids, approximately 1% of wet weight of the lens, are found mainly in cell membranes. The major (50–60% of all lipids of the lens) is cholesterol (Jacob *et al*., 1999; Girao *et al*., 1999; VanMarle and Vrensen, 2000).

## Factors implicated in cataract

There is a coincident dehydration of the proteins and the lens itself. Together with modification of the protein and other constituents, these changes result in less flexibility upon aging. As the lens ages, the proteins are photooxidatively damaged, aggregate, and accumulate in lens opacities. Dysfunction of the lens due to opacification is called cataract. The term “*age-related cataract”* is used to distinguish lens opacification associated with old age from opacification associated with other causes, such as *congenital* and metabolic disorders, e.g. diabetes or galactosemia (Jacques and Taylor, 1991; Taylor and Nowell, 1997).

Half-lives of many of the lens proteins are measured in decades. The sunlight and oxygen that the lens is exposed to are associated with extensive damage to the long-lived lens proteins and other constituents. With progressive damage, the altered proteins accumulate, aggregate, and precipitate in opacities, or cataracts. The young lens has substantial reserves of antioxidants (e.g. vitamins C and E, carotenoids and glutathione (GSH) and antioxidant enzymes (e.g. superoxide dismutase (SOD), catalase (CAT), and glutathione reductase/peroxidase (GSR/GPx)) that may prevent damage. Proteolytic enzymes, called proteases, may selectively remove obsolete proteins and provide a second level of defense. Compromises of function of the lens upon aging are associated with and may be causally related to depleted or diminished primary antioxidant reserves, antioxidant enzyme capabilities, and diminished secondary defenses such as proteases. Environmental stress such as smoking and excessive UV-light exposure, appear to provide an additional oxidative challenge associated with the depletion of antioxidants as well as with enhanced risk for cataract (Taylor and Nowell, 1997). Other risk factors for cataract formation include diabetes, galactosemia, electromagnetic radiation, life-threatening diarrhea, renal failure and many drugs (Cerami and Crabbe, 1986). The most common drugs and related compounds implicated in cataract formation, in humans and in experimental animals, are given in Table 1.

**Table 1:** Drugs and related compounds implicated in cataract formation (Cerami and Crabbe, 1986).

**Cataract in experimental animals**
cyanate, methylisocyanate, N-methyl-N-nitrosourea, bisulphan, dinitrophenol, 3-aminotriazole, naphthalene, triparanol and other inhibitors of cholesterol synthesis, ecothiopate iodide (phospholine iodide) and other cholinesterase inhibitors, diquat, chloroquine, chlopromazine and phenotiazines, adrenaline and morphine, steroids, bleomycin
**Cataract in humans**
barbiturates, alcohol, dinitrophenol, triparanol and other inhibitors of cholesterol synthesis, cholinesterase inhibitors, phenotiazines and major tranquilizers, diuretics, steroids

## Crystallins and their role in maintaining tranparency

Crystallins are the major structural proteins in the lens accounting for up to 90% of total soluble protein. There are three distinct families of crystallins: α-, β- and γ-crystallins. Among these, the α- and β-crystallins exist as oligomers, whereas the γ-crystallin is a monomer. Their structure, stability and short-range interactions are thought to contribute to lens transparency. The human lens is also susceptible to age-related degenerative changes such as accumulation of insoluble proteins and oxidative damage and hence senile cataracts are the most common form of cataract (Harding, 1991; Ponce *et al*., 2006; Takemoto and Sorensen, 2008).

α-Crystallin, a member of the small heat shock protein family, constitutes a major portion of the eye lens cytoplasm. It constitutes up to 50% of the total protein (Bloemendal *et al*., 2004). α-Crystallin monomer has a molecular weight of 20,000. In humans, the lenticular α-crystallin exists as a heterooligomer of the approximate molecular weight of 800,000 with two subunits, αA and αB occurring in a stoichiometry of 3:1 (de Jong *et al*., 1998). αA-Crystallin appears to be largely lens-specific, whereas αB-crystallin is also expressed in other tissues such as heart, skeletal muscle, kidney, and brain. Increased levels of αB-crystallin have been observed in many neurodegenerative disorders, tumors and diabetic conditions (Klemenz *et al*., 1991; Kumar *et al*., 2005a). Both of these proteins are known for their chaperone activity as evident from suppression of protein aggregation. They presumably protect other lens proteins from the adverse effects of heat, chemicals, and UV irradiation. In addition to providing refractive properties to the eye lens, α-crystallins are instrumental in maintaining transparency of the lens with their chaperone-like activity (Harding, 1991; Bera *et al*., 2002; Horwitz, 2003; Surolia *et al*., 2008). Recently it has been reported that some low molecular weight peptides found in aged and cataractous lens bind and reduce the chaperone-like activity of α-crystallin (Rao *et al*., 2008).

The proteins of the eye lens are extremely long-lived and there is virtually no protein turnover. This provides great opportunities for post-translational modifications (PTMs) to occur, most of which lead to aggregation and this process is further accelerated due to various physiological, environmental and genetic factors that predispose lens to cataract formation (Harding, 1991). PTMs were found to induce changes of higher order structure of lens proteins related to opacification (Zhang *et al*., 2001; MacCoss *et al*., 2002; Fujii *et al*., 2004; Ponce *et al*., 2006; Wilmarth *et al*., 2006; Zhang *et al*., 2007; Takemoto and Sorensen, 2008; Kanamoto *et al*., 2009; Hains and Truscott, 2010).

Multiple biochemical mechanisms are involved in the opacification of the lens and they have been thoroughly described: i) non-enzymatic glycation (Thorpe and Baynes, 1996; Lapolla *et al*., 2006; Padival and Nagaraj, 2006), ii) oxidative stress (Spector, 2000; Paron *et al*., 2004; Niwa, 2007), iii) polyol pathway (Jedzniak *et al*., 1981; Kador *et al*., 2007; Lorenzi, 2007) and iv) activation of calpain proteases (Chandrasekher and Cenedella, 1993; Ma *et al*., 1999; Nakamura *et al*., 2000).

## Different experimental approaches in cataractogenic research

Especially in rodents the observation of eye lens proteome related changes have been studied preferentially on the aging model of cataract (Cenedella, 1998; Lampi *et al*., 2002; Ueda *et al*., 2002; Descamps *et al*., 2005) or on the hereditary cataract model (Fujii *et al*., 2004). However, only few of the research articles were found to be related to diabetic cataractous state in particular (Satake *et al*., 2003; Kumar *et al*., 2005a; Kumar *et al*., 2005b).

Although dogs (Kador *et al*., 2007; Gift *et al*., 2009) and rabbits (Cheng, 2002; Babizhayev *et al*., 2009) might be commonly used, rodents still remain the most common experimental animals used to study the mechanisms of cataract formation. Several experimental treatments aimed at inducing cataracts in rats include streptozotocin-induced diabetes (Kyselova *et al*., 2005a; Kyselova *et al*., 2005b), galactose feeding (Huang *et al*., 1990; Huang *et al*., 2000), ionizing radiation (Worgul *et al*., 1996), inhibition of cholesterol synthesis and steroid treatment (Dickerson *et al*., 1997), overdose of selenite (Shearer *et al*., 1997), and finally culture with oxidants or calcium ionophore (Chandrasekher and Cenedella, 1993; Fukiage *et al*., 1997; Nakamura *et al*., 1999; Mitton *et al*., 1999).

In most of these models, covalent modification of crystallins, followed by phase separation of lens cytosol and formation of water-insoluble aggregates, may play important roles in opacification. Some of the modifications detected in rat crystallins that could contribute to insolubilization are mixed disulfide formation (Lou *et al*., 1995; Kyselova *et al*., 2005c), glycation (Swamy-Mruthinti *et al*., 1996), cross-linking by UV-light (Dillon *et al*., 1989), transglutaminase (Groenen *et al*., 1994) or disulfides (Ozaki *et al*., 1987), phosphorylation (Ito *et al*., 1999) and proteolysis (David *et al*., 1993).

The ocular lens would appear to be an ideal organ for maintaining culture conditions because of lacking blood vessels and nerves. The lens *in vivo* obtains its nutrients and eliminates waste products via diffusion with the surrounding fluids.

Lens opacification observed *in vivo* can be mimicked *in vitro* by the addition of a cataractogenic agent to the culture medium – e.g. galactose (Saxena *et al*., 1996) or high glucose (Padival and Nagaraj, 2006; Dickerson *et al*., 1995; Olofsson *et al*., 2007; Son *et al*., 2007). Parenthetically, the lenses from various species have been incubated successfully since the middle of the last century (Kuck, 1970). Different research groups utilized rat lenses in organ culture as a model system for studying the effects of various stresses on the lens, mechanisms of cataract formation, and for screening potential anti-cataract agents (Kinoshita, 1974; Spector *et al*., 1998; Zigler *et al*., 2003; Ghosh and Zigler, 2005). In certain instances lens opacification induced *in vivo* by administration of a particular cataractogenic agent can be mimicked *in vitro* by addition of the same agent to the culture medium – e.g. naphthalene (Xu *et al*., 1992; Lee and Chung, 1998), selenite (Biju *et al*., 2007a), transforming growth factor-β (Hales *et al*., 1995), methylglyoxal (Shamsi *et al*., 2000) or high glucose (Padival and Nagaraj, 2006; Dickerson *et al*., 1995; Olofsson *et al*., 2007; Son *et al*., 2007; Devamanoharan and Varma, 1995). Further, agents which prevent such cataracts *in vivo* may also be effective in culture (Son *et al*., 2007; Zigler *et al*., 2003; Chandra *et al*., 2002). Thus, there is good evidence to support the idea that lenses in culture can be an effective model for the lens *in vivo*.

## Two-faced biological function of selenium

Selenium (Se) is an essential trace element for humans, animals, and some bacteria. It is important for many cellular processes: it is dietarily essential, being specifically incorporated into the active sites of several known proteins or enzymes as the amino acid selenocysteine (Letavayova *et al*., 2006). It is pharmacologically active and at supranutritional dietary levels can prevent the development of many cancers, thus demonstrating chemoprevention and/or carcinostatic activities (Rayman, 2005). Moreoever, Se functions in the body as an antioxidant, it is involved in thyroid hormone metabolism, redox reactions, reproduction, and immune function (Rayman, 2000; Combs *et al*., 2009). Selenium has however been shown to induce wide-spread oxidative stress in biological systems (Manikandan *et al*., 2009). Ironically, it forms an important part of biological defense, being the key component of selenoproteins, such as GPx, selenoprotein P and thioredoxin reductases (Stadtman, 1991). Indeed, Se has been shown to protect against cadmium-mediated apoptosis by regulating reactive oxygen species (ROS) generation and mitochondria linked signaling pathways (Zhou *et al*., 2009).

Selenium has been linked to regulatory functions in cell growth, survival and cytotoxicity, as well as transformations possibly involving redox regulation, chemical toxicity (Zhou *et al*., 2009). Some reports showed that Se can ameliorate the kidney damage induced by HgCl_2_ injection (El-Shenawy and Hassan, 2008), and Se also had hepato-protective effects against cadmium toxicity in rats (Newairy *et al*., 2007). These reports showed that protection of Se treatment might be associated with recovering inhibition of GPx and thioredoxin reductase activities, decreasing free radical-mediated lipid peroxidation and GSH regeneration (Gan *et al*., 2002).

Selenium intake is mainly in the form of organic compounds ingested in grains, meat, yeast, and vegetables (Cao *et al*., 2004). The Food and Nutrition Board, USA Institute of Medicine (2000) considered the estimated safe and adequate daily intake for Se to be 50–200 µg, with 55 µg/day being the Recommended Dietary Allowance (RDA) for Se for both men and women. The upper Se levels (the highest daily level of Se intake that is likely to pose no risk of adverse health effects in almost all individuals) were fixed at 400 µg Se/day. The No Observed Adverse Effect Level (NOAEL) of dietary Se was estimated to be 1,540–1,600 µg/day (Whanger, 2004).

At higher dietary levels, many Se compounds can become toxic (Spallholz, 1994). All these attributes of Se mainly depend upon the concentration, the chemical form and metabolic activity of the compound (El-Bayoumy, 2001; Whanger, 2004). A common specific characteristic of Se compounds expressing the carcinostatic activity and toxicity *in vitro* and *in vivo* is their interaction with thiols and the generation of free radical species (Kramer and Ames, 1988; Spallholz, 1997). In accordance with the prooxidant activities of Se, the higher doses of some Se compounds have the potential to induce DNA damage (Lu *et al*., 1994, 1995; Zhou *et al*., 2003; Reid *et al*., 2004; Wycherly *et al*., 2004; Waters *et al*., 2005).

Chronic exposure in humans or animals results in selenosis (Goldhaber, 2003). Selenosis is characterized by hair loss, fingernail changes and brittleness, gastrointestinal disturbances, skin rash, garlic breath, and abnormal functioning of the nervous system. Other related toxic effects are disruption of endocrine function, of synthesis of thyroid hormones and growth hormones, and an insulin-like growth factor metabolism (Navarro-Alarcon and Cabrera-Vique, 2008). The mechanism of Se toxicity has not been clarified but mostly attributed to its ability to induce oxidative stress both *in vitro* and *in vivo* (Kitahara *et al*., 1993; Yan and Spallholz, 1993; Manikandan *et al*., 2009; Valdiglesias *et al*., 2009)

## Selenite model of cataractogenesis

The selenite cataract model is the most commonly used as it partially mimics senile nuclear cataract in humans. This chapter will briefly outline the methodological particulars and will try to explain possible mechanisms of cataract formation induced by sodium selenite. Since the model has been used by several investigators to screen a variety of agents having anti-cataract potential, their representatives will be outlined.

### Experimental approaches *in vivo*

Selenite-overdose cataract is an extremely rapid and convenient model of nuclear cataracts in rats *in vivo*. Sodium selenite is a cataractogenic agent commonly used in experimental studies since 1978 (Ostadalova *et al*., 1978). Selenite cataract is usually produced by a single subcutaneous injection of 19–30 µM/kg body weight of sodium selenite (Na_2_SeO_3_) into suckling rats of 10–14 days of age, definitely before the completion of the critical maturation period of the lens at approximately 16 days of age (Shearer *et al*., 1997). Repeated injections of smaller doses of selenite (Huang *et al*., 1992) or oral administration (Shearer *et al*., 1983) are also cataractogenic.

Severe, bilateral nuclear cataracts are produced within 4–6 days. Precursor stages include: posterior subcapsular cataract (day 1), swollen fibers (day 2–3), and perinuclear refractile ring (day 3). Although the model has been used extensively as a model for nuclear cataract, a transient cortical cataract also forms 15–30 days after injection (Shearer *et al*., 1992). The cortical cataract then clears after several months, but the nuclear cataract is permanent.

Similarly, Andreson *et al*. (1988) observed that after a single injection of an overdose of sodium selenite at 30 µM/kg b.w., the nuclear cataract appeared rapidly within 3–5 days after injection and was permanent, while cortical cataract developed 15–30 days after injection and cleared within a few months. The selenite cortical cataract appeared to arise from early epithelial damage which interrupted normal fibergenesis and interfered with normal ion control, resulting in an influx of water, cellular destruction and opacity. Remarkably, selenite cortical cataract spontaneously cleared after several months, restoring essentially normal cells to the epithelium and outer and mid-cortex. Major mechanisms for clearing probably involved: (1) removal of damaged proteins from the lens by extensive proteolysis; and (2) replacement of fibers by resumption of normal fibergenesis. Their data emphasized the remarkable reparative potential of the lens to restore clarity after severe damage.

### Experimental approaches *in vitro*

Usually Wistar rats of either sex in the weight range of 100 to 200 g can be used for the study. When these rats are killed the eyes are enucleated without delay. The lenses are carefully dissected out from a posterior approach to avoid damage. Next, according to Biju *et al*. (2007b), the lenses are cultured as organs in M-199 medium with HEPES buffer, supplemented with 10% fetal calf serum (FCS), 100 U/ml penicillin, 0.1 mg/ml streptomycin, and 0.25 µg/ml amphotericin under 5% CO_2_ at 37 °C in a CO_2_ incubator. Selenite medium is prepared by adding sodium selenite to the medium to give a final concentration of 0.1 mM. Lenses are maintained in a 24-well culture plate with 2 ml medium/well and one lens/well for five days. Lenses developing opacification in the first 24 h are discarded because of high suspicion for ruptures during preparation. Then, as early as twenty-four hours of incubation in the presence of sodium selenite lenses result in a dense cortical vacuolization and opacification.

### Sodium selenite safety concerns

Sodium selenite is a salt, a colorless solid, and the most common water-soluble selenium compound. It has the formulas Na_2_SeO_3_ and Na_2_SeO_3_(H_2_O)_5_. Respectively, these are the anhydrous salt and its pentahydrate. The latter salt is the more common one.

The LD50 (intravenous) for sodium selenite in rats is listed in the Material Safety Data Sheet (Sigma) as 3 mg/kg. The cataractogenic dose is 2.4 mg/kg (Shearer *et al*., 1997). This dose does not cause observable effects in suckling rats, except for occasional skin lesions at the injection site. Furthermore, sodium selenite is excreted in urine, feces, and expired air. Precautions are needed for animal handlers and for disposal of sodium selenite injection solutions, animal cadavers and wastes. A significant portion of this dose is retained in the cadavers because Se is incorporated into tissue proteins (Shearer and Hadjimarkos, 1973).

### Mechanism of cataract formation induced by sodium selenite

As indicated above, in both experimental approaches, either *in vivo* or *in vitro*, sodium selenite manifests its effect on lens by inducing primarily oxidative stress in lens tissue. However, its exact mode of action is still open to debate. Fris *et al*. (2006) hypothesized that the formation of selenite-induced nuclear cataract is a result of GSH loss from the lens. Thereafter, the capacity of GSH to buffer the oxidation/reduction status of lens metabolism is diminished, and the sensitivity of rat lenses to oxidative stress is enhanced. As a consequence of selenite treatment, the metabolic profile of the rat lens is dramatically changed. Between 24 and 96 hours after selenite injection, the total pool of free amino acids (excluding taurine) is elevated and remains increased for 8 days (Mitton *et al*., 1997). The water content in the lenses remains stable over this period, so that changes in concentrations of specific amino acids reflect firstly altered metabolism. Furthermore, the energy metabolism in the lens is impaired. Selenite reaction with GSH increases requirements for energy compounds like reduced nicotinamide adenine dinucleotide phosphate (Mitton *et al*., 1997) and the enhanced demand is met by activation of the pentose phosphate pathway in the lens (Kinoshita and Wachtl, 1958), resulting in accumulation of three-carbon metabolites.

**Figure 2:**
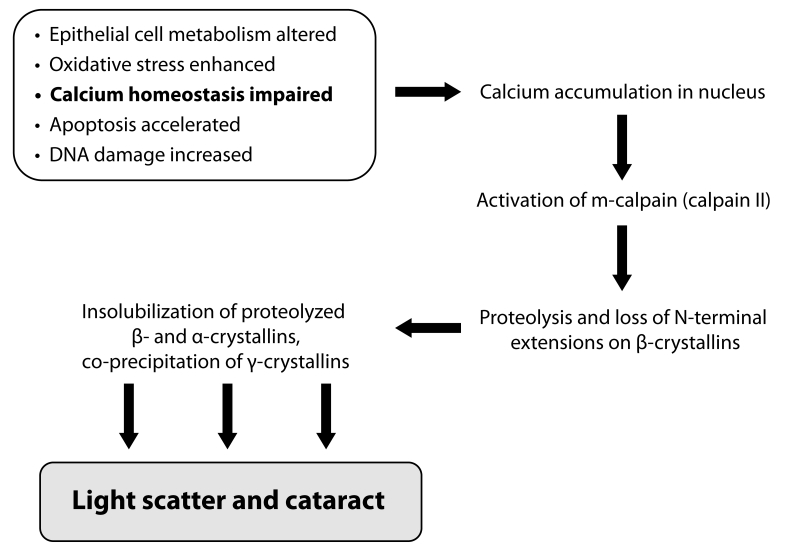
Mechanims of selenite-induced nuclear cataract formation (Shearer *et al*., 1997-modified).

The selenite-induced nuclear cataract formation is caused by various contributing mechanisms summarized in Figure 2, including calpain-induced hydrolysis and precipitation of lenticular proteins. Calpains (EC 3.4.22.17) are a family of non-lysosomal cysteine proteases with a neutral pH optimum and a requirement of calcium for activation.

Documented changes in the metabolism of lenticular epithelial cells during selenite cataractogenesis (usually well before the appearance of any visible opacity) include suppression of mitosis and entry of epithelial cells into prophase, nuclear fragmentation (Anderson *et al*., 1986), decreased rate of epithelial cell differentiation (Cenedella, 1987), increased damage to DNA (Huang *et al*., 1990), and loss of calcium homeostasis (Wang *et al*., 1993). In fact, normal and selenite cataractous lenses have been found to differ in the expression of 91 different genes, with the most obvious differences being noted in the cytochrome c oxidase subunit I (COX-I) and gastric inhibitory polypeptide genes (expression of these genes was found to be decreased in selenite cataractous lenses) and in the early growth response protein-1 (EGR-1) gene (expression of this gene was found to be increased in selenite cataractous lenses; Nakajima *et al*., 2002). Interestingly, two of these three genes, namely EGR-1 and COX-I, are involved in apoptosis (Nakajima *et al*., 2002).

The accelerated apoptosis (programmed cell death) can be regarded as an additional mechanism for selenite cataract. For example, apoptosis in lens epithelial cells could lead to loss of lens homeostasis, allowing calcium influx into the underlying fiber cells. Recently apoptosis in lens epithelium was proposed as contributing to calcimycin-induced cataract (Li *et al*., 1995a) and to the UVB-induced cataracts (Michael *et al*., 1998). During normal development of the eye, apoptosis is required for the separation of the lens from the future corneal epithelium (Garcia-Porrero *et al*., 1979; Schook, 1980) and for removal of the *tunica vasculosa lentis* and the anterior pupillary membrane (Lang *et al*., 1994; Latker and Kuwabara, 1981). Removal of cells is beneficial to the organism, but apoptosis may be triggered prematurely, as in neurons subjected to ischemic conditions resulting from heart attack or stroke (Vaux and Strasser, 1996; Hetts, 1998) or during retinal ganglion cell death in glaucoma (Quigley *et al*., 1995). Calpain and other proteases, such as caspases, are involved in cell death in other tissues (Wang *et al*., 1996), and these enzymes might likewise be activated after the oxidative damage to the lens caused by selenite. The experimental data of Tamada *et al*. (2000) indicated that apoptosis was increased in selenite cataract and that m-calpain and caspase activity were activated, thus apoptosis may be a fairly early event in selenite cataract. Moroeover, apoptosis in lens epithelial cells has been reported in other cataract models. Li *et al*. (1995b) demonstrated that lens epithelial cells from human cataract patients exhibited much higher rates of apoptosis than age-matched controls. The lens is reliant on the epithelial layer of cells for maintaining metabolic homeostasis (Spector, 1991). In cultured rat lenses subjected to peroxide, lens epithelial apoptosis preceded cataract formation. Similar inducers of cataract formation, such as UV irradiation (Michael *et al*., 1998), are also known to induce apoptosis. It is possible that diverse types of cataracts may be initiated through a common mechanism involving apoptosis in the lens epithelium.

Of the all above-mentioned biochemical processes contributing to cataractogenesis, ionic homeostasis seems to be an integrating factor for maintenance of lens transparency (Biju *et al*., 2007b). Loss of Ca^2+^ homeostasis has been implicated in most types of cataract (Duncan *et al*., 1993; Duncan *et al*., 1994). Levels of Ca^2+^ are maintained in the sub-µM range in the cytoplasm by membrane Ca^2+^ pumps (Galvan and Louis, 1988), plasma membrane Na^+^/Ca^2+^ exchangers (Churchill and Louis, 1999), and endoplasmic reticulum Ca^2+^ pumps (Shearer *et al*., 1997). Increased Ca^2+^ uptake performed in connection with selenite cataractogenesis, was found to be highest in the nucleus (Hamakubo *et al*., 1986). An important consequence of calcium elevation in lens is the activation of calpains (Shearer *et al*., 1997). Studies on experimental cataract have demonstrated calpain-induced proteolysis of β-crystallin as a major mechanism in lens maturation as well as cataractogenesis (David *et al*., 1994). Lp82 is the dominant isoform of calpain in rodent lens, suggesting that it may be responsible for the proteolysis attributed to calpains in experimental cataract. Alterations to membrane proteins, lipid integrity, and the consequent increase of membrane ion permeability of the lens fiber cells have been reported in different pathological conditions (Jacques and Chylack, 1991; Stitt, 2001). Thus, selenite-induced oxidative stress and the subsequent loss of Ca^2+^ homeostasis are responsible for the activation of lens calpains, which results in proteolytic precipitation and aggregation of soluble proteins to insoluble proteins.

### Potential anti-cataract agents tested in the selenite model

Selenite-induced oxidative stress mediated cataractogenesis has been shown to be prevented by antioxidative agents such as caffeic acid phenethyl ester (Doganay *et al*., 2002), 2-ketoglutarate (Varma and Hedge, 2004) and extract of *Ocimum sanctum* (Gupta *et al*., 2005). In these studies, the putative anti-cataractogenic effect is thought to be due to the maintenance of normal antioxidant levels and preventing alterations of lens protein. Gupta *et al*. (2002) evaluated the anticataract potential of polyphenolic compounds present in green tea (*Camellia sinensis*). Their results suggest that green tea possesses significant anticataract potential and acts primarily by preserving the antioxidant defense system. Another nutritional antioxidant, lycopene, was tested by Gupta *et al*. (2003) and again was shown to protect against experimental cataract development by virtue of its antioxidant properties.

Resveratrol, a phytoalexin produced naturally by several plants, was able to suppress selenite-induced oxidative stress and cataract formation in rats (Doganay *et al*., 2006). Its protective effect was supported by the finding of higher GSH and lower malondialdehyde (MDA) levels in lens and erythrocytes.

Study of Yağci *et al*. (2006) with the rat selenite cataract model strongly supported the activity of melatonin as an endogenous antioxidant and anticataract agent: in the melatonin-treated group, the lens and serum levels of the lipid peroxidation marker MDA and oxidative stress indicators as xanthine oxidase and protein carbonyls were significantly decreased. On the contrary, the levels of antioxidant enzymes SOD and CAT were significantly increased when compared with the selenite non-treated group.

The study of Biju *et al*. (2007b) was aimed to test Drevogenin D, a triterpenoid aglycone isolated from a woody climbing plant *Dregea volubilis*, as a potential therapeutic agent against oxidative stress-induced cataract. The results obtained indicated that Drevogenin D treatment was effective in protecting the lens proteins by controlling stress-induced protein oxidation, maintenance of Ca^2+^ ATPase activity, calcium accumulation, lipid peroxidation, and prevention of calpain activation. Among potential anti-cataract agents, positive outcomes for good antioxidant activities were found for acetyl-L-carnitine (Elanchezhian *et al*., 2007) and ellagic acid – a naturally-occurring polyphenol (Sakthivel *et al*., 2008).

In 2009, the rising number of studies using selenite models and screening for anti-cataract agents indicates the increasing interest of investigators for the given topic. A herbal remedy was recommended for treatment by Javadzadeh *et al*. (2009a): in their study intraperitoneal injection of aqueous garlic extract into rats appeared to effectively prevent selenite-induced cataract *in vivo*. Surprisingly for onion, a further work of Javadzadeh *et al*. (2009b) testing instillation of onion juice into the rat eyes (one drop of 50% diluted fresh juice of crude onion, applied every 8 hours into the right eye for 14 days) also showed effective prevention of selenite-induced cataract formation. This was associated with increased total antioxidant level and the activities of SOD and GPx within the lens. The prevention of selenite-induced cataractogenesis was also declared by rutin (Isai *et al*., 2009a), by an extract of the oyster mushroom *Pleurotus Ostreatus* (Isai *et al*., 2009b), by curcumin and aminoguanidin (Manikandan *et al*., 2009), by N-acetyl cysteine (Aydin *et al*., 2009) and finally by a topical use of coenzyme Q10-loaded liposomes coated with trimethyl chitosan (Zhang and Wang, 2009).

Tamada *et al*. (2001) tested the calpain inhibitor SJA6017 which ameliorated *in vivo* selenite cataract formation in rats, thus stressing the significance of calcium homeostasis for manitaining healthy lens conditions. Recently Rooban *et al*. (2009) tested phytochemical antioxidants isolated from *Vitex negundo*. Using the selenite-induced cataract model, they assessed the efficacy of flavonoids tested in preventing changes associated with oxidative stress, loss of calcium homeostasis, calpain activation and protein insolubilization in the lens. The activities of SOD, CAT, Ca^2+^ ATPase, concentration of reduced GSH and protein sulfhydryl content were significantly increased in treated groups compared to the non-treated one. Moreover, decreased activities of calpains, lower concentration of calcium and thiobarbituric acid reactive substances (TBARS) were observed in treated groups as compared to the non-treated one.

The differential expression of apoptotic genes has been demonstrated in selenite-induced cataract (Nakajima *et al*., 2002). Recently, Elanchezhian *et al*. (2010) carried out an experiment to investigate the possibility of acetyl-L-carnitine (ALCAR) to prevent selenite-induced cataractogenesis by regulating the expression of antioxidant (CAT) and apoptotic (caspase-3, EGR-1 and COX-I) genes. The expression of lenticular caspase-3 and EGR-1 genes appeared to be up-regulated, as inferred by detecting increased mRNA transcript levels, while that of COX-I and CAT genes appeared to be down-regulated (lowered mRNA transcript levels) in the lenses of cataract-untreated rats. However, in rats treated with ALCAR, the lenticular mRNA transcript levels were maintained at near normal (control) levels. Their results suggest an original approach how to prevent selenite-induced cataractogenesis by affecting the abnormal expression of lenticular genes governing apoptosis.

## Concluding remarks towards selenite model relevancy

Oxidative stress as a contributing mechanism is a key factor in modelling cataractogenesis. Most of the effects of anti-cataract compounds tested (see chapter above) were elucidated by their antioxidant actions. Thus, the model is relevant since selenite is a strong oxidant. Selenite-induced opacification of lens may partially mimic oxidant exposures commonly present in the environment of a man (e.g. from sunlight) and/or occurring at pathophysiological conditions (e.g. diabetes, aging etc.).

According to Doganay *et al*. (2002) selenite cataract has many general similarities to human cataract, e.g. lipid membrane vesicles formation, increased level of calcium, elevated amount of insoluble proteins, enhanced proteolysis, decreased amount of water soluble proteins and declined level of GSH. Major dissimilarities are also present contrary to human cataract: no high molecular weight covalent aggregates or increased disulfide formation. Selenite cataract appears to be dominated by rapid calpain-induced proteolytic precipitation (Shearer *et al*., 1997), while human senile cataract may be caused by oxidative stress over a long period. Currently, the best conclusion about the relevancy of selenite cataract model to human cataract may be that selenite cataract is a useful biological model for initial drug testing. However, important differences between human and selenite cataracts have to be taken into consideration when scaling up the verdicts.

## References

[CIT0001] Anderson RS, Shearer TR, Claycomb CK (1986). Selenite-induced epithelial damage and cortical cataract. Curr Eye Res.

[CIT0002] Anderson RS, Trune DR, Shearer TR (1988). Histologic changes in selenite cortical cataract. Invest Ophthalmol Vis Sci.

[CIT0003] Augusteyn RC (2007). Growth of the human eye lens. Mol Vis.

[CIT0004] Aydin B, Yagci R, Yilmaz FM, Erdurmus M, Karadağ R, Keskin U, Durmus M, Yigitoglu R (2009). Prevention of selenite-induced cataractogenesis by N-acetylcysteine in rats. Curr Eye Res.

[CIT0005] Babizhayev MA, Guiotto A, Kasus-Jacobi A (2009). N-Acetylcarnosine and histidyl-hydrazide are potent agents for multitargeted ophthalmic therapy of senile cataracts and diabetic ocular complications. J Drug Target.

[CIT0006] Bassnett S (2002). Lens organelle degradation. Exp Eye Res.

[CIT0007] Bassnett S (2009). On the mechanism of organelle degradation in the vertebrate lens. Exp Eye Res.

[CIT0008] Bera S, Thampi P, Cho WJ, Abraham EC (2002). A positive charge preservation at position 116 of αA-crystallin is critical for its structural and functional integrity. Biochemistry.

[CIT0009] Biju PG, Rooban BN, Lija Y, Devi VG, Sahasranamam V, Abraham A (2007b). Drevogenin D prevents selenite-induced oxidative stress and calpain activation in cultured rat lens. Mol Vis.

[CIT0010] Biju PG, Devi VG, Lija Y, Abraham A (2007a). Protection against selenite cataract in rat lens by drevogenin D, a triterpenoid aglycone from *Dregea volubilis*. J Med Food.

[CIT0011] Bloemendal H, Bloemendal H (1981). The lens proteins. Molecular and Cellular Biology of the Eye Lens.

[CIT0012] Bloemendal H, de Jong W, Jaenicke R, Lubsen NH, Slingsby C, Tardieu A (2004). Ageing and vision: structure, stability and function of lens crystallins. Prog Biophys Mol Biol.

[CIT0013] Bosch AM (2006). Classical galactosaemia revisited. J Inherit Metab Dis.

[CIT0014] Cao S, Durrani FA, Rustum YM (2004). Selective modulation of the therapeutic efficacy of anti-cancer drugs by selenium containing compounds against human tumor xenografts. Clin Cancer Res.

[CIT0015] Cenedella RJ (1987). Direct chemical measurement of DNA synthesis and net rates of differentiation of rat lens epithelial cells *in vivo*: applied to the selenium cataract. Exp Eye Res.

[CIT0016] Cenedella RJ (1998). Prenylation of proteins by the intact lens. Invest Ophthalmol Vis Sci.

[CIT0017] Cerami A, Crabbe J (1986). Recent advances in ocular cataract research. TIPS Reviews.

[CIT0018] Chandra D, Ramana KV, Wang L, Christensen BN, Bhatnagar A, Srivastava SK (2002). Inhibition of fiber cell globulization and hyperglycemia-induced lens opacification by aminopeptidase inhibitor bestatin. Invest Ophthalmol Vis Sci.

[CIT0019] Chandrasekher G, Cenedella RJ (1993). Calcium activated proteolysis and protein modification in the U18666A cataract. Exp Eye Res.

[CIT0020] Cheng HM (2002). Water diffusion in the rabbit lens *in vivo*. Dev Ophthalmol.

[CIT0021] Chikamoto N, Chikama T, Yamada N, Nishida T, Ishimitsu T, Kamiya A (2009). Efficacy of substance P and insulin-like growth factor-1 peptides for preventing postsurgical superficial punctate keratopathy in diabetic patients. Jpn J Ophthalmol.

[CIT0022] Churchill GC, Louis CF (1999). Imaging of intracellular calcium stores in single permeabilized lens cells. Am J Physiol.

[CIT0023] Combs GF, Midthune DN, Patterson KY, CanWeld WK, Hill AD, Levander OA, Taylor PR, Moler JE, Patterson BH (2009). Effects of selenomethionine supplementation on selenium status and thyroid hormone concentrations in healthy adults. Am J Clin Nutr.

[CIT0024] David LL, Azuma M, Shearer TR (1994). Cataract and the acceleration of calpain-induced β-crystallin insolubilization occurring during normal maturation of rat lens. Invest Ophthalmol Vis Sci.

[CIT0025] David LL, Shearer TR, Shih M (1993). Sequence analysis of lens β-crystallins suggests involvement of calpain in cataract formation. J Biol Chem.

[CIT0026] de Jong WW, Caspers GJ, Leunissen JA (1998). Genealogy of the α-crystallin-small heat-shock protein superfamily. Int J Biol Macromol.

[CIT0027] Descamps FJ, Martens E, Proost P, Starckx S, van den Steen PE, van Damme J, Opdenakker G (2005). Gelatinase B/matrix metalloproteinase-9 provokes cataract by cleaving lens βB1-crystallin. FASEB J.

[CIT0028] Devamanoharan PS, Varma SD (1995). Inhibition of polyol formation in rat lens by verapamil. J Ocul Pharmacol Ther.

[CIT0029] Dickerson JE, Lou MF, Gracy RW (1995). The culture of rat lenses in high sugar media: effect on mixed disulfide levels. Curr Eye Res.

[CIT0030] Dickerson JE, Dotzel E, Clark AF (1997). Steroid-induced cataract: new perspective from *in vitro* and lens culture studies. Exp Eye Res.

[CIT0031] Dillon J, Roy D, Spector A, Walker ML, Hibbard LB, Borkman RF (1989). UV laser photodamage to whole lenses. Exp Eye Res.

[CIT0032] Doganay S, Borazan M, Iraz M, Cigremis Y (2006). The effect of resveratrol in experimental cataract model formed by sodium selenite. Curr Eye Res.

[CIT0033] Doganay S, Turkoz Y, Evereklioglu C, Er H, Bozaran M, Ozerol E (2002). Use of caffeic acid phenethyl ester to prevent sodium selenite-induced cataract in rat eyes. J Cataract Refr Surg.

[CIT0034] Duncan G, Webb SF, Dawson AP, Bootman MD, Elliott AJ (1993). Calcium regulation in tissue-cultured human and bovine lens epithelial cells. Invest Ophthalmol Vis Sci.

[CIT0035] Duncan G, Williams MR, Raich RA (1994). Calcium, cell signaling and cataract. Prog Ret Eye Res.

[CIT0036] Elanchezhian R, Ramesh E, Sakthivel M, Isai M, Geraldine P, Rajamohan M, Jesudasan CN, Thomas PA (2007). Acetyl-L-carnitine prevents selenite-induced cataractogenesis in an experimental animal model. Curr Eye Res.

[CIT0037] Elanchezhian R, Sakthivel M, Geraldine P, Thomas PA (2010). Regulatory effect of acetyl-l-carnitine on expression of lenticular antioxidant and apoptotic genes in selenite-induced cataract. Chem Biol Interact.

[CIT0038] El-Bayoumy K (2001). The protective role of selenium on genetic damage and on cancer. Mutat Res.

[CIT0039] El-Shenawy SM, Hassan NS (2008). Comparative evaluation of the protective effect of selenium and garlic against liver and kidney damage induced by mercury chloride in the rats. Pharmacol Rep.

[CIT0040] Fan X, Zhang J, Theves M, Strauch C, Nemet I, Liu X, Qian J, Giblin FJ, Monnier VM (2009). Mechanism of lysine oxidation in human lens crystallins during aging and in diabetes. J Biol Chem.

[CIT0041] Food and Nutrition Board – USA Institute of Medicine (2000). Dietary References Intakes for Vitamin C, Vitamin E, Selenium and Carotenoids.

[CIT0042] Fris M, Tessem MB, Sather O, Midelfart A (2006). Biochemical changes in selenite cataract model measured by high-resolution MAS (1)H NMR spectroscopy. Acta Ophthalmol Scand.

[CIT0043] Fujii N, Takeuchi N, Fujii N, Tezuka T, Kuge K, Takata T, Kamei A, Saito T (2004). Comparison of post-translational modifications of αA-crystallin from normal and hereditary cataract rats. Amino Acids.

[CIT0044] Fukiage C, Azuma M, Nakamura Y, Tamada Y, Nakamura M, Shearer TR (1997). SJA6017, a newly synthesized peptide aldehyde inhibitor of calpain: amelioration of cataract in cultured rat lenses. Biochim Biophys Acta.

[CIT0045] Galvan A, Louis CF (1988). Calcium regulation by lens plasma membrane vesicles. Arch Biochem Biophys.

[CIT0046] Gan L, Liu Q, Xu HB, Zhu YS, Yang XL (2002). Effects of selenium overexposure on glutathione peroxidase and thioredoxin reductase gene expressions and activities. Biol Trace Elem Res.

[CIT0047] Garcia-Porrero JA, Collado JA, Ojeda JL (1979). Cell death during detachment of the lens rudiment from ectoderm in the chick embryo. Anat Rec.

[CIT0048] Ghosh MP, Zigler JS (2005). Lack of fiber cell induction stops normal growth of rat lenses in organ culture. Mol Vis.

[CIT0049] Gift BW, English RV, Nadelstein B, Weigt AK, Gilger BC (2009). Comparison of capsular opacification and refractive status after placement of three different intraocular lens implants following phacoemulsification and aspiration of cataracts in dogs. Vet Ophthalmol.

[CIT0050] Girao H, Mota C, Pereira P (1999). Cholesterol may act as an antioxidant in lens membranes. Curr Eye Res.

[CIT0051] Goldhaber SB (2003). Trace element risk assessment: essentiality *vs*. toxicity. Regul Toxicol Pharmacol.

[CIT0052] Graw J (2003). The genetic and molecular basis of congenital eye defects. Nat Rev Genet.

[CIT0053] Groenen PJ, Grootjans JJ, Lubsen NH, Bloemendal H, de Jong WW (1994). Lys-17 is the amine-donor substrate site for transglutaminase in βA3-crystallin. J Biol Chem.

[CIT0054] Gupta SK, Trivedi D, Srivastava S, Joshi S, Halder N, Varma SD (2003). Lycopene attenuates oxidative stress-induced experimental cataract development: an *in vitro* and *in vivo* study. Nutrition.

[CIT0055] Gupta SK, Srivastava S, Trivedi D, Joshi S, Halder N (2005). *Ocimum sanctum* modulates selenite-induced cataractogenic changes and prevents rat lens opacification. Curr Eye Res.

[CIT0056] Gupta SK, Halder N, Srivastava S, Trivedi D, Joshi S, Varma SD (2002). Green tea (*Camellia sinensis*) protects against selenite-induced oxidative stress in experimental cataractogenesis. Ophthalmic Res.

[CIT0057] Hains P, Truscott RJ (2010). Age-dependent deamidation of life-long proteins in the human lens. Invest Ophthalmol Vis Sci.

[CIT0058] Hales AM, Chamberlain CG, McAvoy JW (1995). Cataract induction in lenses cultured with transforming-growth-factor- β. Invest Ophthalmol Vis Sci.

[CIT0059] Hamakubo T, Kannagi R, Murachi T, Matus A (1986). Distribution of calpains I and II in rat brain. J Neurosci.

[CIT0060] Hammond CJ, Duncan DD, Snieder H, de Lange M, West SK, Spector TD, Gilbert CE (2001). The heritability of age-related cortical cataract: the twin eye study. Invest Ophthalmol Vis Sci.

[CIT0061] Harding JJ (1991). Post-translational modification of lens proteins in cataract. Lens Eye Toxic Res.

[CIT0062] Hetts SW (1998). To die or not to die: An overview of apoptosis and its role in disease. JAMA.

[CIT0063] Horwitz J (2003). Alpha-crystallin. Exp Eye Res.

[CIT0064] Huang LL, Hess JL, Bunce GE (1990). DNA damage, repair and replication in selenite-induced cataract in rat lens. Curr Eye Res.

[CIT0065] Huang LL, Zhang C-Y, Hess JL, Bunce GE (1992). Biochemical changes and cataract formation in lenses from rats receiving multiple, low doses of sodium selenite. Exp Eye Res.

[CIT0066] Huang FY, Ho Y, Shaw TS, Chuang SA (2000). Functional and structural studies of α-crystallin from galactosemic rat lenses. Biochem Biophys Res Commun.

[CIT0067] Huang WQ, Zhang JP, Fu JSC (1990). Differential effects of galactose-induced cataractogenesis on the soluble crystallins of rat lens. Exp Eye Res.

[CIT0068] Isai M, Elanchezhian R, Sakthivel M, Chinnakkaruppan A, Rajamohan M, Jesudasan CN, Thomas PA, Geraldine P (2009b). Anti-cataractogenic effect of an extract of the oyster mushroom, *Pleurotus ostreatus*, in an experimental animal model. Curr Eye Res.

[CIT0069] Isai M, Sakthivel M, Ramesh E, Thomas PA, Geraldine P (2009a). Prevention of selenite-induced cataractogenesis by rutin in Wistar rats. Mol Vis.

[CIT0070] Ito H, Iida K, Kamei K, Iwamoto I, Inaguma Y, Kato K (1999). αB-crystallin in the rat lens is phosphorylated at an early post-natal age. FEBS Lett.

[CIT0071] Jacob RF, Cenedella RJ, Mason RP (1999). Direct evidence for immiscible cholesterol domains in human ocular lens fiber cell plasma membranes. J Biol Chem.

[CIT0072] Jacques PF, Chylack LT (1991). Epidemiologic evidence of a role for the antioxidant vitamins and carotenoids in cataract prevention. Am J Clin Nutr.

[CIT0073] Jacques PF, Taylor A, Bendich A, Butterworth CE (1991). Micronutrients and age-related cataracts. Micronutrients in health and in disease prevention.

[CIT0074] Jaffe NS, Horwitz J, Podos SM, Yanoff M (1991). Lens and Cataract. Text Book of Ophthalmology 3.

[CIT0075] Javadzadeh A, Ghorbanihaghjo A, Arami S, Rashtchizadeh N, Mesgari M, Rafeey M, Omidi Y (2009a). Prevention of selenite-induced cataractogenesis in Wistar albino rats by aqueous extract of garlic. J Ocul Pharmacol Ther.

[CIT0076] Javadzadeh A, Ghorbanihaghjo A, Bonyadi S, Rashidi MR, Mesgari M, Rashtchizadeh N, Argani H (2009b). Preventive effect of onion juice on selenite-induced experimental cataract. Indian J Ophthalmol.

[CIT0077] Jedziniak JA, Chylack LT, Cheng HM, Gillis MK, Kalustian AA, Tung WH (1981). The sorbitol pathway in the human lens: aldose reductase and polyol dehydrogenase. Invest Ophthalmol Vis Sci.

[CIT0078] Johnson GJ, Foster A, Johnson GJ, Minassian DC, Weale RA, West SK (2004). Prevalence, incidence and distribution of visual impairment. The epidemiology of eye disease.

[CIT0079] Kador PF, Takahashi Y, Akagi Y, Blessing K, Randazzo J, Wyman M (2007). Age-dependent retinal capillary pericyte degeneration in galactose-fed dogs. J Ocul Pharmacol Ther.

[CIT0080] Kanamoto T, Souchelnytskyi N, Kiuchi Y (2009). Functional proteomics of failed filtering blebs. Mol Vis.

[CIT0081] Kinoshita JH, Wachtl C (1958). A study of the C14-glucose metabolism of the rabbit lens. J Biol Chem.

[CIT0082] Kinoshita JH (1974). Mechanisms initiating cataract formation. Proctor Lecture. Invest Ophthalmol.

[CIT0083] Kitahara J, Seko Y, Imura N (1993). Possible involvement of active oxygen species in selenite toxicity in isolated rat hepatocytes. Arch Toxicol.

[CIT0084] Klemenz R, Fröhli E, Aoyama A, Hoffmann S, Simpson RJ, Moritz RL, Schäfer R (1991). αB-crystallin accumulation is a specific response to Ha-ras and v-mos oncogene expression in mouse NIH 3T3 fibroblasts. Mol Cell Biol.

[CIT0085] Kramer GF, Ames BN (1988). Mechanisms of mutagenicity and toxicity of sodium selenite (Na_2_SeO_3_) in *Salmonella typhimurium*. Mutat Res.

[CIT0086] Kuck JFR, Graymore CN (1970). Clinical constituents of the lens, metabolism of the lens, cataract formation. Biochemistry of the eye.

[CIT0087] Kumar PA, Haseeb A, Suryanarayana P, Ehtesham NZ, Reddy GB (2005a). Elevated expression of αA- and αB-crystallins in streptozotocin-induced diabetic rat. Arch Biochem Biophys.

[CIT0088] Kumar PA, Reddy PY, Srinivas PN, Reddy GB (2009). Delay of diabetic cataract in rats by the anti-glycating potential of cumin through modulation of α-crystallin chaperone activity. J Nutr Biochem.

[CIT0089] Kumar PA, Suryanarayana P, Reddy PY, Reddy GB (2005b). Modulation of α-crystallin chaperone activity in diabetic rat lens by curcumin. Mol Vis.

[CIT0090] Kupfer C (1984). The conquest of cataract: A global challenge. Trans Ophthal Soc UK.

[CIT0091] Kuszak JR, Tasman W, Jaeger EA (1990). Embryology and anatomy of the lens. Clinical Ophthalmology.

[CIT0092] Kyselova Z, Gajdosik A, Gajdosikova A, Ulicna O, Mihalova D, Karasu C, Stefek M (2005b). Effect of the pyridoindole antioxidant stobadine on development of experimental diabetic cataract and on lens protein oxidation in rats: comparison with vitamin E and BHT. Mol Vis.

[CIT0093] Kyselova Z, Garcia SJ, Gajdosikova A, Gajdosik A, Stefek M (2005a). Temporal relationship between lens protein oxidation and cataract development in streptozotocin-induced diabetic rats. Physiol Res.

[CIT0094] Kyselova Z, Krizanova L, Soltes L, Stefek M (2005c). Electrophoretic analysis of oxidatively modified eye lens proteins *in vitro*: implications for diabetic cataract. J Chromatogr A.

[CIT0095] Lampi KJ, Shih M, Ueda Y, Shearer TR, David LL (2002). Lens proteomics: analysis of rat crystallin sequences and two-dimensional electrophoresis map. Invest Ophthalmol Vis Sci.

[CIT0096] Lang R, Lustig M, Francois F, Sellinger M, Plesken H (1994). Apoptosis during macrophage-dependent ocular tissue remodeling. Development.

[CIT0097] Lapolla A, Fedele D, Seraglia R, Traldi P (2006). The role of mass spectrometry in the study of non-enzymatic protein glycation in diabetes: an update. Mass Spectrom Rev.

[CIT0098] Latker CH, Kuwabara T (1981). Regression of the *tunica vasculosa lentis* in the postnatal rat. Invest Ophthalmol Vis Sci.

[CIT0099] Lee AY, Chung SS (1998). Involvement of aldose reductase in naphthalene cataract. Invest Ophthalmol Vis Sci.

[CIT0100] Letavayova L, Vlckova V, Brozmanova J (2006). Selenium: from cancer prevention to DNA damage. Toxicology.

[CIT0101] Li WC, Kuszak JR, Dunn K, Wang RR, Ma W, Wang GM, Spector A, Leib M, Cotliar AM, Weiss M, Espy J, Howard G, Farris RL, Auran J, Donn A, Hofeldt A, Mackay C, Merrian J, Mittl R, Smith TR (1995b). Lens epithelial cell apoptosis appears to be a common cellular basis for non-congenital cataract development in human and animals. J Cell Biol.

[CIT0102] Li WC, Kuszak JR, Wang GM, Wu ZQ, Spector A (1995a). Calcimycin-induced lens epithelial cell apoptosis contributes to cataract formation. Exp Eye Res.

[CIT0103] Lorenzi M (2007). The polyol pathway as a mechanism for diabetic retinopathy: attractive, elusive, and resilient. Exp Diabetes Res.

[CIT0104] Lou MF, Xu GT, Cui XL (1995). Further studies on the dynamic changes of glutathione and protein-thiol mixed disulfides in H_2_O_2_-induced cataract in rat lenses: distributions and effect of aging. Curr Eye Res.

[CIT0105] Lovicu FJ, Robinson ML, Lovicu FJ, Robinson ML (2004). Development of the Ocular Lens.

[CIT0106] Lu J, Kaeck M, Jiang C, Wilson AC, Thompson HJ (1994). Selenite induction of DNA strand breaks and apoptosis in mouse leukemic L1210 cells. Biochem Pharmacol.

[CIT0107] Ma H, Hata I, Shih M, Fukiage C, Nakamura Y, Azuma M, Shearer TR (1999). Lp82 is the dominant form of calpain in young mouse lens. Exp Eye Res.

[CIT0108] MacCoss MJ, McDonald WH, Saraf A, Sadygov R, Clark JM, Tasto JJ, Gould KL, Wolters D, Washburn M, Weiss A, Clark JI, Yates JRI (2002). Shotgun identification of protein modifications from protein complexes and lens tissue. Proc Nat Acad Sci USA.

[CIT0109] Manikandan R, Thiagarajana R, Beulaja S, Chindhud S, Mariammale K, Sudhandiranc G, Arumugama M (2009). Anti-cataractogenic effect of curcumin and aminoguanidine against selenium-induced oxidative stress in the eye lens of Wistar rat pups: An *in vitro* study using isolated lens. Chem Biol Interact.

[CIT0110] Michael R, Vrensen GF, van Marle J, Gan L, Soderberg PG (1998). Apoptosis in the rat lens after *in vivo* threshold dose ultraviolet irradiation. Invest Ophthalmol Vis Sci.

[CIT0111] Minassian DC, Reidy A, Desai P, Farrow S, Vafidis G, Minassian A (2000). The deficit in cataract surgery in England and Wales and the escalating problem of visual impairment: epidemiological modeling of the population dynamics of cataract. Br J Ophthalmol.

[CIT0112] Mitton KP, Hess JL, Bunce GE (1997). Free amino acids reflect impact of selenite-dependent stress on primary metabolism in rat lens. Curr Eye Res.

[CIT0113] Mitton KP, Linklater HA, Dzialoszynski T, Sanford SE, Starkey K, Trevithick JR (1999). Modelling cortical cataractogenesis 21: in diabetic rat lenses taurine supplementation partially reduces damage resulting from osmotic compensation leading to osmolyte loss and antioxidant depletion. Exp Eye Res.

[CIT0114] Nakajima T, Nakajima E, Fukiage C, Azuma M, Shearer TR (2002). Differential gene expression in the lens epithelial cells from selenite injected rats. Exp Eye Res.

[CIT0115] Nakamura Y, Fukiage C, Azuma M, Shearer TR (1999). Oxidation enhances calpain-induced turbidity in young rat lenses. Curr Eye Res.

[CIT0116] Nakamura Y, Fukiage C, Shih M, Ma H, David LL, Azuma M, Shearer TR (2000). Contribution of calpain Lp82-induced proteolysis to experimental cataractogenesis in mice. Investig Ophthalmol Vis Sci.

[CIT0117] Navarro-Alarcon M, Cabrera-Vique C (2008). Selenium in food and the human body: a review. Sci Total Environ.

[CIT0118] Newairy AA, El-Sharaky AS, Badreldeen MM, Eweda SM, Sheweita SA (2007). The hepatoprotective effects of selenium against cadmium toxicity in rats. Toxicology.

[CIT0119] Niwa T (2007). Protein glutathionylation and oxidative stress. J Chromatogr B Analyt Technol Biomed Life Sci.

[CIT0120] Olofsson EM, Marklund SL, Behndig A (2007). Glucose-induced cataract in CuZn-SOD null lenses: an effect of nitric oxide?. Free Radic Biol Med.

[CIT0121] Ostadalova I, Babicky A, Obenberger J (1978). Cataract induced by administration of a single dose of sodium selenite to suckling rats. Experientia.

[CIT0122] Ozaki Y, Mizuno A, Itoh K, Iriyama K (1987). Inter- and intra-molecular disulfide bond formation and related structural changes in the lens proteins. A Raman spectroscopic study *in vivo* of lens aging. J Biol Chem.

[CIT0123] Padival S, Nagaraj RH (2006). Pyridoxamine inhibits Maillard reactions in diabetic rat lenses. Ophthalmic Res.

[CIT0124] Paron I, D'Elia A, D'Ambrosio C, Scaloni A, D'Aurizio F, Prescott A, Damante G, Tell G (2004). A proteomic approach to identify early molecular targets of oxidative stress in human epithelial lens cells. Biochem J.

[CIT0125] Ponce A, Sorensen C, Takemoto L (2006). Role of short-range protein interactions in lens opacifications. Mol Vis.

[CIT0126] Quigley HA, Nickells RW, Kerrigan LA, Pease ME, Thibault DJ, Zack DJ (1995). Retinal ganglion cell death in experimental glaucoma and after axotomy occurs by apoptosis. Invest Ophthalmol Vis Sci.

[CIT0127] Rao G, Santhoshkumar P, Sharma KK (2008). Anti-chaperone βA3/A1(102-117) peptide interacting sites in human αB-crystallin. Mol Vis.

[CIT0128] Rayman MP (2000). The importance of selenium to human health. Lancet.

[CIT0129] Rayman MP (2005). Selenium in cancer prevention: a review of the evidence and mechanism of action. Proc Nutr Soc.

[CIT0130] Reid ME, Stratton MS, Lillico AJ, Fakih M, Natarajan R, Clark LC, Marshall JR (2004). A report of high-dose selenium supplementation: response and toxicities. J Trace Elem Med Biol.

[CIT0131] Robman L, Taylor H (2005). External factors in the development of cataract. Eye (Lond).

[CIT0132] Rooban BN, Lija Y, Biju G, Sasikala V, Sahasranamam V, Abraham A (2009). *Vitex negundo* attenuates calpain activation and cataractogenesis in selenite models. Exp Eye Res.

[CIT0133] Sakthivel M, Elanchezhian R, Ramesh E, Isai M, Jesudasan CN, Thomas PA, Geraldine P (2008). Prevention of selenite-induced cataractogenesis in Wistar rats by the polyphenol, ellagic acid. Exp Eye Res.

[CIT0134] Satake M, Dmochowska B, Nishikawa Y, Madaj J, Xue J, Guo Z, Reddy DV, Rinaldi PL, Monnier VM (2003). Vitamin C metabolomic mapping in the lens with 6-deoxy-6-fluoro-ascorbic acid and high-resolution 19F-NMR spectroscopy. Invest Ophthalmol Vis Sci.

[CIT0135] Saxena P, Saxena AK, Monnier VM (1996). High galactose levels *in vitro* and *in vivo* impair ascorbate regeneration and increase ascorbate-mediated glycation in cultured rat lens. Exp Eye Res.

[CIT0136] Schook P (1980). Morphogenetic movements during the early development of the chick eye: An ultrastructual and spatial study. Acta Morphol Neerl Scand.

[CIT0137] Shamsi FA, Sharkey E, Creighton D, Nagaraj RH (2000). Maillard reactions in lens proteins: methylglyoxal-mediated modifications in the rat lens. Exp Eye Res.

[CIT0138] Shearer TR, Anderson RS, Britton JL (1983). Influence of selenite and fourteen trace elements on cataractogenesis in the rat. Invest Ophthalmol Vis Sci.

[CIT0139] Shearer TR, David LL, Anderson RS, Azuma M (1992). Review of selenite cataract. Curr Eye Res.

[CIT0140] Shearer TR, Hadjimarkos DM (1973). Comparative distribution of 75 Se in the hard and soft tissues of mother rats and their pups. J Nutr.

[CIT0141] Shearer TR, Ma H, Fukiage C, Azuma M (1997). Selenite nuclear cataract: review of the model. Mol Vis.

[CIT0142] Shih M, Ma H, Nakajima E, David LL, Azuma M, Shearer TR (2006). Biochemical properties of lens-specific calpain Lp85. Exp Eye Res.

[CIT0143] Son HY, Kim H, H Kwon Y (2007). Taurine prevents oxidative damage of high glucose-induced cataractogenesis in isolated rat lenses. J Nutr Sci Vitaminol.

[CIT0144] Spallholz JE (1994). On the nature of selenium toxicity and carcinostatic activity. Free Radic Biol Med.

[CIT0145] Spallholz JE (1997). Free radical generation by selenium compounds and their prooxidant toxicity. Biomed Environ Sci.

[CIT0146] Spector A (2000). Review: oxidative stress and diseases. J Ocul Pharmacol Ther.

[CIT0147] Spector A, Sies H (1991). The lens and oxidative stress. Oxidative Stress, Oxidants and Antioxidants.

[CIT0148] Spector A, Kuszak JR, Ma W, Wang RR, Ho Y, Yang Y (1998). The effect of photochemical stress upon the lenses of normal and glutathione peroxidase-1 knockout mice. Exp Eye Res.

[CIT0149] Stadtman TC (1991). Biosynthesis and function of selenocysteine-containing enzymes. J Biol Chem.

[CIT0150] Stitt AW (2001). Advanced glycation: an important pathological event in diabetic and age-related ocular disease. Br J Ophthalmol.

[CIT0151] Surolia I, Sinha S, Sarkar DP, Reddy PY, Reddy GB, Surolia A (2008). Concurrence of Danish dementia and cataract: insights from the interactions of dementia associated peptides with eye lens α-crystallin. PLoS One.

[CIT0152] Swamy-Mruthinti S, Green K, Abraham EC (1996). Inhibition of cataracts in moderately diabetic rats by aminoguanidine. Exp Eye Res.

[CIT0153] Takemoto L, Sorensen CM (2008). Protein-protein interactions and lens transparency. Exp Eye Res.

[CIT0154] Tamada Y, Fukiage C, Nakamura Y, Azuma M, Kim YH, Shearer TR (2000). Evidence for apoptosis in the selenite rat model of cataract. Biochem Biophys Res Commun.

[CIT0155] Tamada Y, Fukiage C, Mizutani K, Yamaguchi M, Nakamura Y, Azuma M, Shearer TR (2001). Calpain inhibitor, SJA6017, reduces the rate of formation of selenite cataract in rats. Curr Eye Res.

[CIT0156] Taylor A, Nowell T (1997). Oxidative stress and antioxidant function in relation to risk for cataract. Adv Pharmacol.

[CIT0157] Theodoropoulou S, Theodossiadis P, Samoli E, Vergados I, Lagiou P, Tzonou A (2010). The epidemiology of cataract: A study in Greece. Acta Ophthalmol.

[CIT0158] Thorpe SR, Baynes JW (1996). Role of the Maillard reaction in diabetes mellitus and diseases of aging. Drugs Aging.

[CIT0159] Ueda Y, Fukiage C, Shih M, Shearer TR, David LL (2002). Mass measurements of C-terminally truncated α-crystallins from two-dimensional gels identify Lp82 as a major endopeptidase in rat lens. Mol Cell Proteomics.

[CIT0160] Valdiglesias V, Pásaro E, Méndez J, Laffon B (2009). *In vitro* evaluation of selenium genotoxic, cytotoxic, and protective effects: a review. Arch Toxicol.

[CIT0161] VanMarle J, Vrensen GF (2000). Cholesterol content of focal opacities and multilamellar bodies in the human lens: filipin cytochemistry and freeze fracture. Ophthalmic Res.

[CIT0162] Varma SD, Hedge KR (2004). Effect of α-ketoglutarate against selenite cataract formation. Exp Eye Res.

[CIT0163] Varma SD (1991). Scientific basis for medical therapy of cataracts by antioxidants. Am J Clin Nutr.

[CIT0164] Vaux DL, Strasser A (1996). The molecular biology of apoptosis. Proc Natl Acad Sci USA.

[CIT0165] Wang Z, Bunce GE, Hess JL (1993). Selenite and Ca^2+^ homeostasis in the rat lens: effect on Ca-ATPase and passive Ca^2+^ transport. Curr Eye Res.

[CIT0166] Wang KK, Nath W, Raser KJ, Hajimohammadreza I (1996). Maitotoxin induces calpain activation in SH-SY5Y neuroblastoma cells and cerebrocortical cultures. Arch Biochem Biophys.

[CIT0167] Waters DJ, Shen S, Glickman LT, Cooley DM, Bostwick DG, Qian J, Combs GF, Morris JS (2005). Prostate cancer risk and DNA damage: translational significance of selenium supplementation in a canine model. Carcinogenesis.

[CIT0168] West SK (2000). Looking forward to 20/20: a focus on the epidemiology of eye diseases. Epidemiol Rev.

[CIT0169] Whanger PD (2004). Selenium and its relationship to cancer: an update. Br J Nutr.

[CIT0170] Wilmarth PA, Tanner S, Dasari S, Nagalla SR, Riviere MA, Bafna V, Pevzner PA, David LL (2006). Age-related changes in human crystallins determined from comparative analysis of post-translational modifications in young and aged lens: does deamidation contribute to crystallin insolubility?. J Proteome Res.

[CIT0171] Worgul BV, Medvedovsky C, Huang Y, Marino SA, Randers-Pehrson G, Brenner DJ (1996). Quantitative assessment of the cataractogenic potential of very low doses of neutrons. Radiat Res.

[CIT0172] Wycherly BJ, Moak MA, Christensen MJ (2004). High dietary intake of sodium selenite induces oxidative DNA damage in rat liver. Nutr Cancer.

[CIT0173] Xu GT, Zigler JS, Lou MF (1992). Establishment of a naphthalene cataract model *in vitro*. Exp Eye Res.

[CIT0174] Yağci R, Aydin B, Erdurmuş M, Karadağ R, Gürel A, Durmuş M, Yiğitoğlu R (2006). Use of melatonin to prevent selenite-induced cataract formation in rat eyes. Curr Eye Res.

[CIT0175] Yan L, Spallholz JE (1993). Generation of reactive oxygen species from the reaction of selenium compounds with thiols and mammary tumor cells. Biochem Pharmacol.

[CIT0176] Zhang J, Wang S (2009). Topical use of Coenzyme Q10-loaded liposomes coated with trimethyl chitosan: tolerance, precorneal retention and anti-cataract effect. Int J Pharm.

[CIT0177] Zhang C, Liu P, Wang N, Li Y, Wang L (2007). Comparison of two tandem mass spectrometry-based methods for analyzing the proteome of healthy human lens fibers. Mol Vis.

[CIT0178] Zhang Z, Smith DL, Smith JB (2001). Multiple separations facilitate identification of protein variants by mass spectrometry. Proteomics.

[CIT0179] Zhou YJ, Zhang SP, Liu CW, Cai YQ (2009). The protection of selenium on ROS mediated-apoptosis by mitochondria dysfunction in cadmium-induced LLCPK1 cells. Toxicol In Vitro.

[CIT0180] Zhou N, Xiao H, Li TK, Nur-E-Kamal A, Liu LF (2003). DNA damage-mediated apoptosis induced by selenium compounds. J Biol Chem.

[CIT0181] Zigler JS, Qin C, Kamiya T, Krishna MC, Cheng Q, Tumminia S, Russell P (2003). Tempol-H inhibits opacification of lenses in organ culture. Free Radic Biol Med.

